# Phosphorus Accumulation and Sorption in Calcareous Soil under Long-Term Fertilization

**DOI:** 10.1371/journal.pone.0135160

**Published:** 2015-08-19

**Authors:** Rui Wang, Shengli Guo, Nana Li, Rujian Li, Yanjun Zhang, Jishao Jiang, Zhiqi Wang, Qingfang Liu, Defeng Wu, Qiqi Sun, Lanlan Du, Man Zhao

**Affiliations:** 1 College of Resources and Environment, Northwest A&F University, Yangling 712100, Shaanxi, China; 2 Institute of Soil and Water Conservation, Northwest A&F University, Yangling 712100, Shaanxi, China; 3 Institute of Soil and Water Conservation, Chinese Academy of Sciences and Ministry of Water Resource, Yangling 712100, Shaanxi, China; Huazhong University of Science and Technology, CHINA

## Abstract

Application of phosphorus (P) fertilizers to P-deficient soils can also result in P accumulation. In this study, soil P status and P uptake by apple trees were investigated in 5-, 10-, and 15-year-old orchards in the semi-arid Loess Plateau, China, and subset soils with different soil P statuses (14–90 Olsen-P mg kg^−1^) were selected to evaluate the characteristic P adsorption. Due to the low P-use efficiency (4–6%), total soil P increased from 540 mg kg^−1^ to 904 mg kg^−1^, Olsen-P ranged from 3.4 mg kg^−1^ to 30.7 mg kg^−1^, and CaCl_2_-P increased from less than 0.1 mg kg^−1^ to 0.66 mg kg^−1^ under continuous P fertilization. The P sorption isotherms for each apple orchard were found to fit the Langmuir isotherm model (*R* ^*2*^ = 0.91–0.98). *K* (binding energy) and *Q*_*m*_ (P sorption maximum) decreased, whereas DPS (degree of phosphorus sorption) increased with increasing P concentration. CaCl_2_-P increased significantly with the increase of Olsen-P, especially above the change point of 46.1 mg kg^−1^. Application of surplus P could result in P enrichment in P-deficient soil which has high P fixation capacity, thus posing a significant environmental risk.

## Introduction

The increasing application of phosphorus (P) fertilizers in recent years has significantly improved soil P status in most regions of the world [1–7]. Intensive agricultural activity has greatly changed P flows globally [8], causing P accumulation in the soil [9]. For instance, the cumulative inputs of P fertilizer and manure for the period 1965–2007 in Europe far exceeded the cumulative P uptake by crops (1,115 kg ha^−1^ *vs*. 360 kg ha^−1^ of cropland) [10]. Although fertilizers play a key role in improving the crop productivity, application of P fertilizers in excess of crop requirements is of environmental concern due to the loss of potentially harmful nutrients to the environment [5]. Continuous addition of P to soil may increase the potential risk for P release and loss because of its finite P holding capacity, even in the case that has extremely high P fixation capacity and low P losses in this type of soil.

Long-term application of surplus P fertilizer may contribute to P enrichment in soil [11,12], decrease P sorption and increase P availability and translocation even in soils with high P sorption capacity [6, 13, 14], thus posing a significant environmental risk. Phosphorus input above the critical limit (change-point) may have high potential for P losses [1, 15–17]. The P sorption capacity of soils which accepted P applications for 25 years was generally lower than that of unfertilized soils [18]. Similarly, the P sorption maximum (*Q*_*m*_) and binding energy (*K*) were consistently smaller with P applied to soils [1], while soil P saturation (DPS) increased [19]. Phosphorus sorption index (PSI) values were lower after high rates of P application [6, 20], Mehlich-3 and M_3_P/PSI increased curvilinearly or linearly with P accumulation [13]. This accumulation can lead to an increase in P leaching into subsurface soil layers [21] and in P transported into surface waters via runoff, thus resulting in accelerated eutrophication of surface waters. Although calcareous soils are common throughout the world, and characterized by high pH and P fixation capacity, continuous P application may increase the potential risk of P losses. It has been shown that unexpected incidental P losses from volcanic soils, which were similar to calcareous soils, threatened water quality in Southern Chile, and such soils were generally thought of as P sinks with high P fixation capacity [6]. Thus, a better understanding of P accumulation and adsorption in calcareous soils after long-term P application is helpful for adjusting fertilization for high crop production and environmental safety.

The objectives of this study are to investigate (i) changes in soil total P (TSP), Olsen-P, and CaCl_2_-P in 5-, 10-, and 15-year-old apple orchards in the semi-arid Loess Plateau, China after long-term application of surplus P fertilizers; (ii) changes in *K*, *Q*_*m*_, and DPS; and (iii) the relationship between *K*, *Q*_*m*_, DPS, Olsen-P and CaCl_2_-P.

## Materials and Methods

### Site description

**Fig 1:**
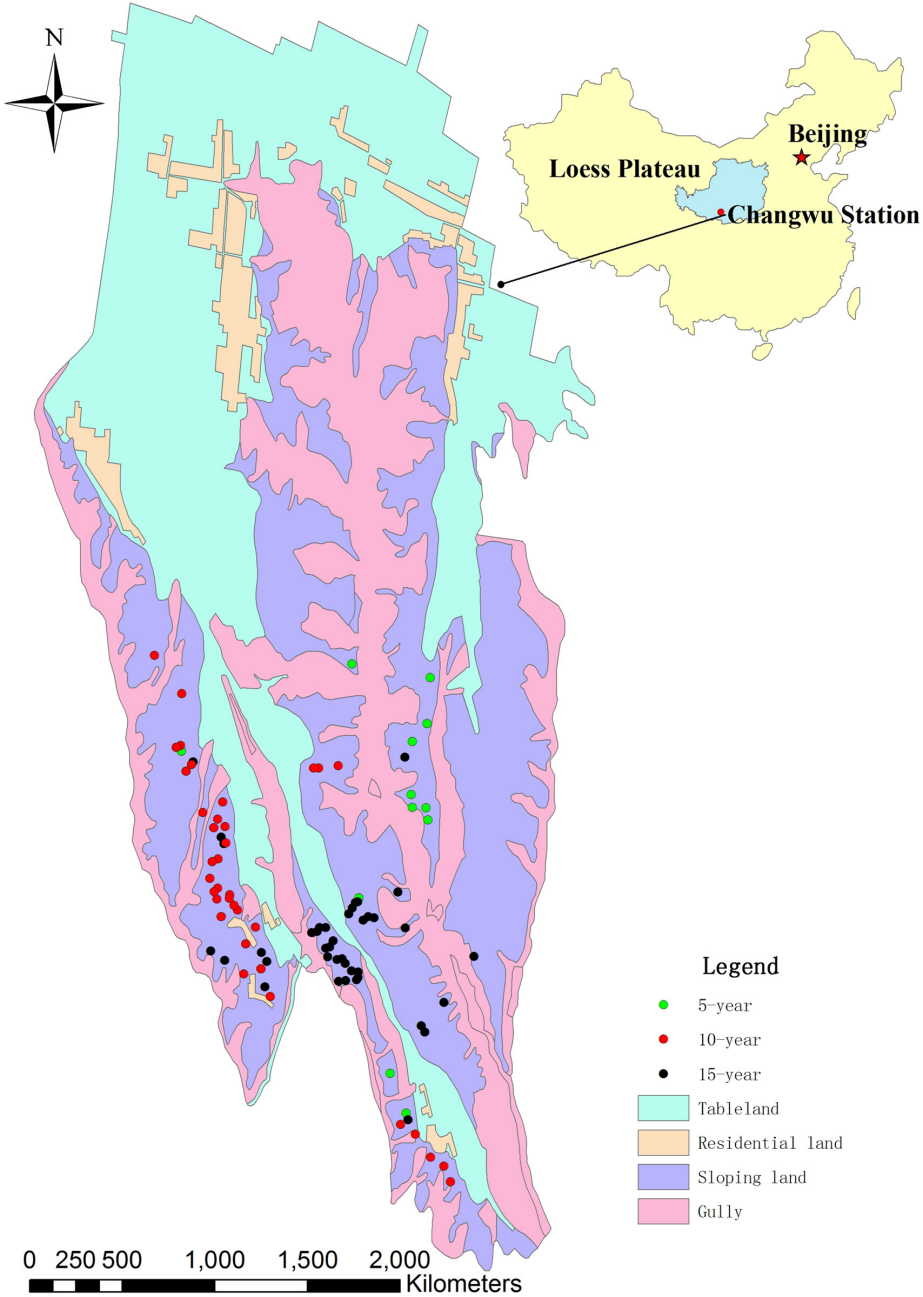
Schematic diagram of sampling points in Wangdonggou watershed.

This study was conducted in a hilly-gully region of Loess Plateau in Wangdonggou watershed, Changwu, Shaanxi, China, (35°12'16'' − 35°16'00'' N, 107°40'30'' − 107°42'30'' E, 1220 m above sea level) (Fig 1). Changwu State Key Agro-Ecological Experimental Station, Chinese Academy of Sciences issued the permission for our experimental sites. The soil erosion modulus is larger than 50 t ha^−1^. Apple orchards have been established in slopeland since 1970s to reduce soil erosion and increase farmer’s income, and now this region has become a typical apple growing region in Loess Plateau.

This region has a semi-arid climate with an average annual temperature of 9.1°C and precipitation of 584 mm (over a recent 30-year period), 55% of which occurs between June and September. Annual sunshine hour is 2230 h, annual solar radiation is 5266 MJ m^−2^, and frost-free period is 194 d. The soil is, derived from wind-deposited loess and, belongs to the Loessal soil group according to the Food and Agriculture Organization and United Nations Education Scientific and Cultural Organization (FAOUNESCO) soil classification system. Soils collected at the study site in 2004 at a depth of 0–20 cm were characterized by: pH 8.4, 24% clay (< 0.002 mm), water-holding capacity 0.29 cm^3^ cm^–3^, organic carbon 6.5 g kg^−1^, total nitrogen 0.8 g kg^−1^, total soil P 640 mg kg^−1^, NH_4_OAc-extractable K 200 mg kg^−1^, CaCO_3_ 105 g kg^−1^, and bulk density 1.3 g cm^–3^, cation exchange capacity (CEC), clay content (< 0.002 mm) 16%, silt content (0.05–0.002 mm) 73%. The groundwater table is 80 m below surface.

### Ethics Statement

There were no specific permits required for the described field studies. We confirmed that the site was not privately owned or protected in any way. The field studies did not involve endangered or protected species.

### Basic information of the apple orchards

Apple tree (*Malus domestica* Borkh) is the most widely cultivated cash crop in Loess Plateau due to its high economic and ecological benefit. Its planting area has increased by 20 times in the past 30 years and now it is estimated to be over 15 million hectare. It is known that the massive amount of inorganic P fertilizer added to soils which has extremely high P fixation capacity remains unavailable for crop uptake. Thus, P deficiency may constitute an important factor contributing to the low crop productivity in Loess Plateau. There is an increasing popularity in the application of P fertilizer since the implementation of land contract responsibility system in China in 1980s. As a result, surplus fertilization is common in some regions. The annual fertilizer inputs reach 750 kg hm^−2^ (P_2_O_5_) or more, soil total P reaches 1400 mg kg^−1^, and Olsen P is more than 100 mg kg^−1^ in some orchards. In this study, 106 apple orchards at the small watershed were selected for soil sampling in September 2004, including 12 5-year-old orchards, 51 10-year-old orchards, and 43 15-year-old orchards (Fig 1). These apple orchards are generally less than 0.3 ha and managed by farm households.

### Sampling Methods

#### Soil sampling

Soil sample was collected from each orchard, and each sample consisted of six subsamples collected at a distance of 1 m from stem at top soil (0–20 cm) by a soil auger (*d* = 4 cm). The reference soils were collected in the grassland which was 1 km away from the apple orchards and received no fertilizer. In addition, soil samples to a depth of 200 cm in 20 cm increments were collected from 12 soil profiles, and there were 3 randomly selected replications for the 5-, 10-, 15-year-old orchards and grassland.

#### Plant sampling

**Fig 2:**
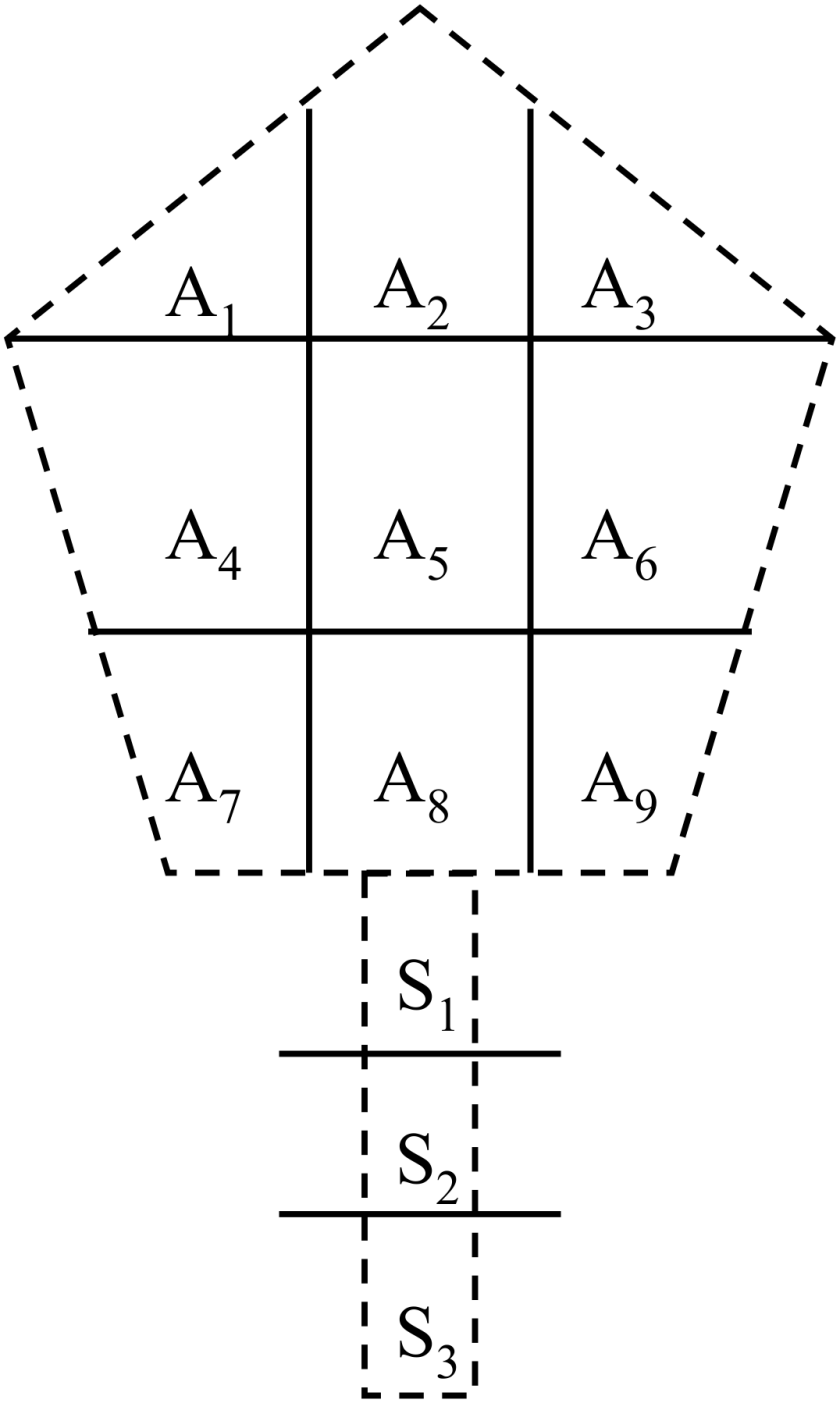
Diagrammatic of the selecting method about sub-samples of apple tree.

Three typical orchards with different ages were selected, and three trees were selected in each orchard at the end of September in 2004 for the measurement of above-ground biomass and nutrient content. For purpose of analysis, the trees were divided into four parts: stem, branches (including central and lateral branches), leaves and fruits. In order to estimate P concentration in different organs, the random with stratified technique sampling was used to select sub-samples. The sample trees were divided into nine units, three different height (upper, middle and lower) at different aspect (inside, middle, and outside), as the Fig 2 shows, sub-samples of central branch, three lateral branches, fruit and leaves which grew well and had no diseases or insect pests was selected by random technique in each unit (A_1_ to A_9_), and sub-samples of stem were composed of S_1_ to S_3_, also by random technique in each unit (Fig 2). Fresh plants were weighted in the field, and sub-samples were washed and oven-dried in laboratory at 80°C for 48 h. Total aboveground biomass was calculated by fresh and dry plant weights, and P uptake for each part was measured by oven-dried samples.

#### Sample analysis

Soil samples were air-dried, sieved to < 2 mm, and digested with HClO_4_-H_2_SO_4_. TSP in bicarbonate and hydroxide extracts were determined by perchloric acid digestion [22]. Available P (Olsen-P) was extracted by NaHCO_3_ at pH = 8.5 [23]. Water-soluble P (WSP) was determined by suspending 1 g of soil in 50 ml of salt solution (0.01 mol L^−1^ CaCl_2_), shaking overhead for 2 h and filtration through a 0.45 μm filter [24, 25]. P in the neutralized extracts and digests was determined by the molybdenum-blue method [24].

The sorption isotherms of P were constructed following the procedures of Nair et al. [26]. Phosphate sorption curves were obtained by shaking 1.0 g of air-dried soil samples in 25 ml of equilibration KH_2_PO_4_ solution containing 0, 5, 10, 30, 50 and 80 μg P ml^−1^ in a 50-ml plastic tube with screw cap for 24 h at 25°C. Then, the solution P concentration was determined by molybdenum-blue method after centrifugation and filtering [24]. Sorption parameters were calculated according to follow Langmuir sorption equation:

$$Q=KQ_{\text{m}}/(1+KC)$$ (1)

where C is the equilibrium concentration of P, *Q* is the amount of P adsorbed, *K* is a constant related to the bonding energy, and *Q*_*m*_ is the P sorption maxima [27].

We then calculated the degree of DPS for the 7 subset soils:

$$DPS(\%)=((Olsen-P)/Q_{\text{m}})\times 100$$ (2)

where *Q*_*m*_ is the P sorption maxima that can be estimated from the linearized Langmuir equation based on the experimental data from P sorption isotherms conducted with 7 soils [28].

Plant samples were ground to < 2 mm and digested in H_2_SO_4_, and four 1.5 ml aliquots of 30% hydrogen peroxide (H_2_O_2_) were added during the digestion. Total P was measured calorimetrically by the vanado-molybdate method.

#### Calculation of P balance

Phosphorus surplus was defined as the difference between P input and output, which represented P stored in soil, and soil P balance was calculated by the following equation [29]:

P surplus=P input − P output (P removed by aboveground parts of plant) (3)

Phosphorus input was mainly from fertilizers, which was equal to the amount of fertilizer applied multiplied by its concentration. The main fertilizers used in these apple orchards included superphosphate, diammonium phosphate and compound fertilizers. Data were obtained from the farmers of the orchards.

### Statistical analysis

Data were processed primarily using the Microsoft Excel spreadsheets. Significance of P balance and soil P accumulation among orchards was tested by the analysis of variance, and the difference was tested by the Least Significant Difference (LSD) using SAS software package. A *p* value less than 0.05 was considered significant. Figures were constructed by SigmPlot 10. The relationship between Olsen-P and CaCl_2_-P was evaluated using the two-segment linear model [17]:

$$y=a_{1}x+b_{1},x\leq T$$ (4)

$$y=a_{2}x+b_{2},x>T$$ (5)

where a_1_, a_2_, b_1_ and b_2_ are the parameters of these equations, and T is the critical level for CaCl_2_-P.

## Results

### Phosphorus inputs and accumulation under long-term phosphate fertilization

P concentration was much higher in the leaves and branches than in the fruits and stems. However, there was no significant difference in P concentration in organs in different apple orchards except the branches. The biomass increased with the age of the orchards. The aboveground biomass (except fruits) was much higher in the 15-year-old apple orchard than in the 5- or the 10-year-old apple orchards (Table 1). Specifically, the aboveground biomass (leaves, branches and stem) in the 15-year-old apple orchard was 1.7–3.2 times that of the 10-year-oldapple orchard, and 2.8–7.2 times that of the 5-year-oldapple orchard, respectively. Fruits in the 15-year-old apple orchard were similar to that in the 10-year-old apple orchard, both of which were much higher than that in the 5-year-old apple orchard. One possible explanation for this is that the 5-year-old apple trees were much younger.

**Table 1:** Phosphorus concentration and aboveground biomass in different organs of apple trees.

Orchard age	Fruit		Leaf		Branches		Stem	
	Concentration	Biomass	Concentration	Biomass	Concentration	Biomass	Concentration	Biomass
5-year	760±20 a	0.3±0.02 b	1380±20 a	0.68±0.12 c	1060±327 a	5.48±1.11 c	480±40 a	2.3±0.84 c
10-year	750±40 a	4.4±1.2 a	1060±90 ba	1.67±0.46 b	957±225 a	9.08±1.96 b	480±70 a	7.11±1.03 b
15-year	770±40 a	4.46±0.97 a	1170±30 a	2.89±0.91 a	793±110 b	15.06±3.2 a	470±20 a	16.63±1.1 a

Note : Phosphorus concentration: mg kg^−1^, aboveground biomass: kg, different letters indicate significant differences among orchard ages (*p*< 0.05).

Total P applied in the 15-year-old apple orchard was about 6.2 and 2.4 times that of 5- and 10-year-old apple orchards. The amount of P removed by apple trees was calculated by summing the nutrient content of stem, branches, leaves and fruits (dry weight multiplied by P concentration per ha), and it was 10.0, 35.3, and 70.4 P kg ha^−1^ for the 5-, 10-, and 15-year-old apple orchards, with a P utilization efficiency of only 4%, 6%, and 5%, respectively (Tables 1 and 2). These results indicated that total P input was greater than P output in this soil-orchard system.

**Table 2:** Phosphorus balance in the three different age apple orchard (P kg ha^−1^).

Orchard age	5-year	10-year	15-year
Inputs	228	585	1404
Outputs			
Fruit	0.4	14.1	28.7
Leaf	3.9	11.2	25.3
Branches	4.8	7.2	9.9
Stem	0.9	2.8	6.5
Total	10.0	35.3	70.4
Balance			
P surplus	218.0	549.7	1333.6

### Phosphorus balance under long-term phosphate fertilization

**Fig 3:**
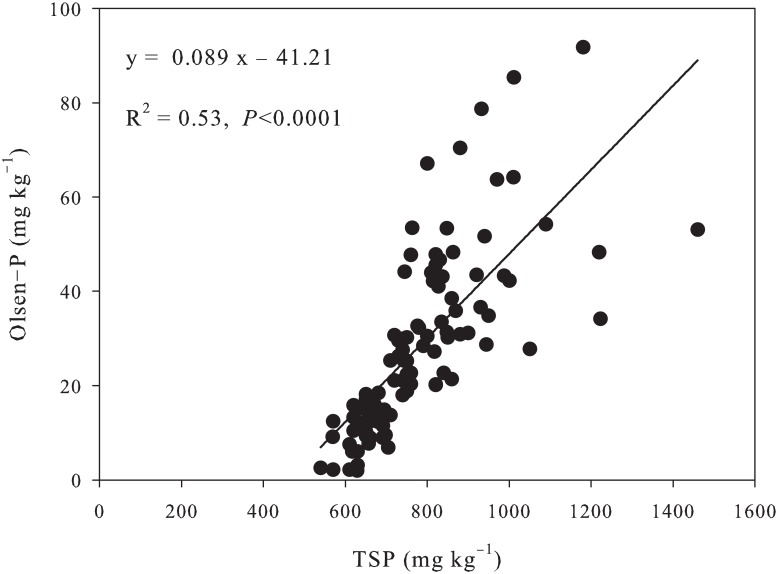
Relationship between total soil P and Olsen-P in P-accumulated soils.

**Fig 4:**
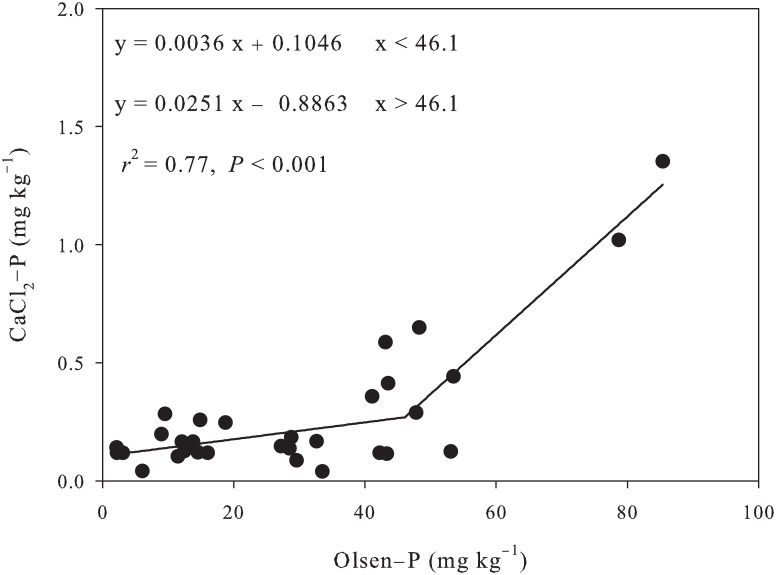
Relationships between CaCl_2_-P and Olsen-P in P-accumulated soils.

The average TSP and Olsen-P of the topsoil (0–20 cm) of grassland was 560 mg kg^−1^ and 3.1 mg kg^−1^, respectively. In comparison, a large amount of P has accumulated in soils of apple orchard under long-term phosphate fertilization (Table 3). TSP was increased by 25% (699.4 mg kg^−1^), 48% (826.3 mg kg^−1^) and 60% (903.5 mg kg^−1^) in 5-, 10-, and 15-year-old apple orchards, respectively. Similarly, Olsen-P was increased by 6 times (19.6 mg kg^−1^), 10 times (30.7 mg kg^−1^), and 12 times (38.9 mg kg^−1^); and CaCl_2_-P was increased from an undetectable level in grassland to 0.66 mg kg^−1^ in 15-year-old orchard (Table 3). In addition, the regression analysis indicated that Olsen-P was linearly related to TSP (*R* ^2^ = 0.53, *P* < 0.0001) (Fig 3), and CaCl_2_-P increased significantly with the increase of Olsen-P, especially above the change point of 46.1 mg kg^−1^ (R^2^ = 0.77, *P* <0.001) (Fig 4). CaCl_2_-P was a major factor for P pollution.

**Table 3:** Effects of the number of years on soil total P, Olsen-P and CaCl_2_-P in orchard.

Orchard age	*N*	TSP	Olsen-P	CaCl_2_-P
5-year	12	699.4±41.4 b	19.6±3.2 c	0.30±0.01c
10-year	51	826.3±157.5 a	30.7±3.6 b	0.40±0.03b
15-year	43	903.5±67.0 a	38.9±5.2 a	0.66±0.03a

Note : Total P, Olsen-P and CaCl_2_-P: mg kg^−1^, Different letters indicate significant differences among orchard ages (*p* < 0.05).

### Changes in *Q*_m_ and DPS in P-accumulated soils

**Fig 5:**
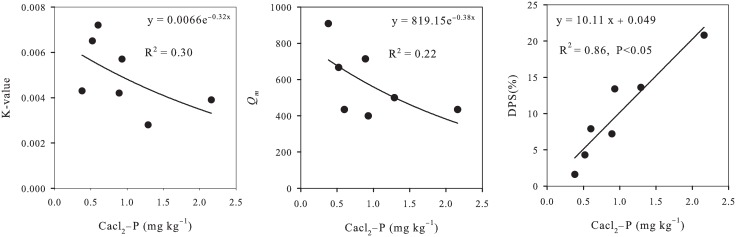
Relationships among K-value, Qm, DPS, and CaCl_2_-P under P-accumulated soils.

The sorption isotherms of each apple orchard were found to fit the Langmuir isotherm model (*R* ^2^ = 0.91–0.98). Fig 5a and 5b showed that the *K* and *Q*_*m*_ values decreased dramatically with increasing P concentration. Average *K* decreased from 0.0072 to 0.0028, and *Q*_*m*_ decreased by 52% from 909 mg kg^−1^ to 435 mg kg^−1^ with the increase of CaCl_2_-P from 0.38 mg kg^−1^ to 2.16 mg kg^−1^. However, DPS increased by about 13 times from 1.6% to 20.8% (Fig 5c). All these results suggested that soil P sorption capacity decreased with increasing P accumulation in soil.

## Discussion

### Phosphorus accumulation in calcareous soil of Loess Plateau

**Fig 6:**
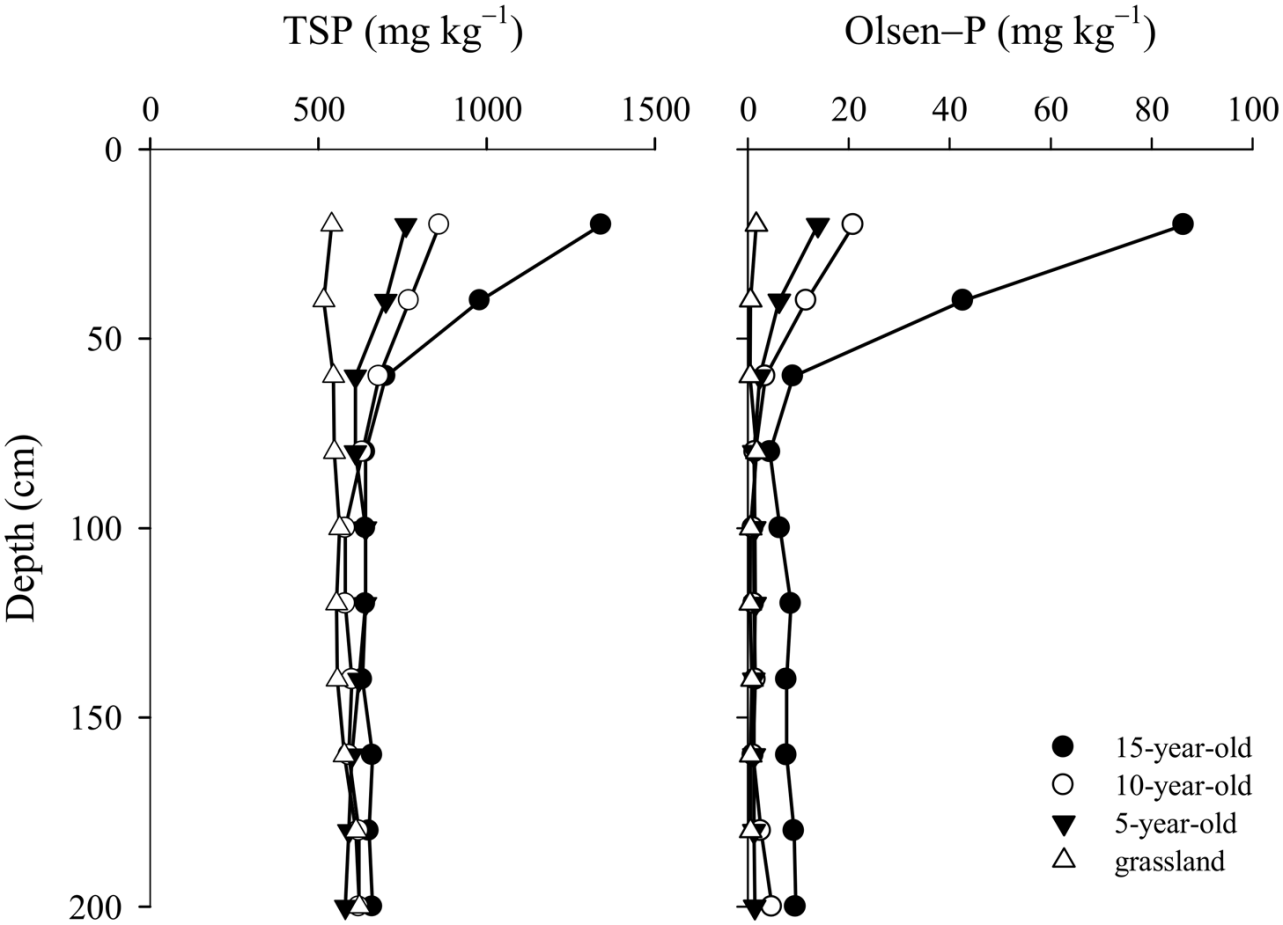
Total P and Olsen-P distribution in the 0 − 200 cm profile under P-accumulated soil.

In China, agricultural fertilizer consumption has been growing at an unprecedented rate since the end of 1980s, and approximately 52.39 million tons of fertilizer was used in 2008, accounting for 31.4% of the global total consumption [30]. In the Loess Plateau, the application of fertilizer has also been on the raise in recent years [31–33], and the annual fertilizer input was about 750 kg hm^−2^ (P_2_O_5_) or more in apple orchards converted from cropland. Calcareous soils in the Loess Plateau have a high TSP [34], but the available P (Olsen-P) in soil is as low as 5.9 mg kg^−1^ [33], which can have a significant effect on crop production in this region. The results of this study showed that P input far exceeded the removal of P in this region, and the use efficiency was low (4% − 6%) because of the high fixation of P [35]. The apple orchard has accumulated 868 kg P ha^−1^ over a period of 15 years, with an average rate of about 58 kg ha^−1^ year^−1^. It was reported that the productive grassland and arable area accumulated an average P surplus of c. 1000 kg ha^−1^ over a period of 65 years in UK [3], net total P accumulation in soils at Crichton ranged from 16 kg ha^−1^ to 232 kg ha^−1^ [1], and the total P concentration (15 cm depth) ranged from 920 mg kg^−1^ without P application to 3750 mg kg^−1^ with 16-year P application in Canada [2]. However, P accumulation in these countries was largely due to the application of manure, especially in areas having intensive livestock production; whereas P accumulation in the Loses Plateau of China was mostly caused by the application of phosphate fertilizers. In general, P accumulation could enhance the translocation of dissolved P from topsoil to subsoil. However, there are few studies estimating the extent of such translocation. In this study, P has accumulated to a depth of 60 cm, and Olsen-P at a depth of 60–200 cm was higher in 15-year-old orchard than in other orchards, indicating that P accumulation resulted in P translocation in soils (Fig 6). The translocation of P from topsoil to subsoil has also been observed in North America and Europe [7]. In the USA, the vertical movement of P in some cultivated profiles occurred to a depth of 40 cm [36], and long-term surplus P fertilization resulted in P accumulation to 75 cm depth in Demark [7].

Soil P sorption has been suggested to be closely related to P accumulation, particularly Olsen-P and CaCl_2_-P. In this study, we determined *K* values, *Q*_*m*_ values and DPS for soils with low, middle, and high P status (Table 4), and the results showed that P sorption decreased as soils become more saturated with P. These parameters were suggested to be related to the heterogeneity of sorption sites, which was defined as any reaction center that could result in the transfer of P from solution to solid phase [37]. These sites also show a reduced affinity for P according to both *K* and *Q*_*m*_ values. Long-term application of surplus P fertilizers may result in P enrichment of the soils [11, 12], saturated soil P sorption sites and decline of P holding capacity. In this study, high P accumulation may contribute to the accelerated translocation of P from topsoil to subsoil. Olsen-P increased by 11 mg kg^−1^ with the increase of TSP from 699 mg kg^−1^ to 826 mg kg^−1^, and by 9 mg kg^−1^ with further increase of TSP from 826 mg kg^−1^ to 904 mg kg^−1^. Because of the high P fixation capacity of soils, vertical movement of P was generally considered of little importance. We found that long-term application of surplus P in the Loess Plateau resulted in P accumulation to a depth of at least 60 cm (Fig 6). Thus, calcareous soil with high fixation in the Loess Plateau has a high risk of P translocation from topsoil to subsoil under long-term application of surplus P, because they will maintain high P concentration in soil.

**Table 4:** Characteristic value of soil P adsorption (*Q* = *Q*_*m*_ *K*C/(1+*k*C)).

Site	Olsen-P	CaCl_2_-P	*K*	*Q*_*m*_	R^2^	DPS
1	14.57	0.38	0.0043	909.1	0.9148	1.6
2	28.75	0.52	0.0065	666.7	0.9530	4.3
3	34.40	0.60	0.0072	434.8	0.9647	7.9
4	51.66	0.89	0.0042	714.3	0.9362	7.2
5	53.78	0.93	0.0057	400.0	0.9621	13.4
6	68.08	1.29	0.0028	500.0	0.9783	13.6
7	90.38	2.16	0.0039	434.8	0.9289	20.8

Note : Olsen-P and CaCl_2_-P: mg kg^−1^, *K*: μmL·g^−1^, *Q*_*m*_: mg·kg^−1^, DPS: %

### Changes in P forms in Loess Plateau and impact of surplus P on the soil nutrient supply

There have been many studies investigating the changes in P forms in Loess Plateau. Guo et al. found that inorganic P increased with the application of P fertilizer for 13 years, inorganic P was 627.8 μg kg^−1^, including Ca_8_-P 86 μg g^−1^, Ca_8_-P 378.6 μg g^−1^, Al-P 32.1 μg g^−1^, Fe-P 39.1 μg g^−1^, O-P 64.6 μg g^−1^ [38]. Lai et al. showed that Ca-P, O-P, Al-P, and Fe-P accounted for approximately 70%, 9%, 6% and 5% of the total inorganic P, respectively, all of which increased with the addition of P fertilizer [39]. In addition, Li et al. showed that surplus P could decrease the content of exchangeable potassium (K) and calcium (Ca), especially exchangeable Ca, while Ca deficiency will lead to apple tree canker which reduces the quality and quantity of apples [40].

### Environmental and agronomic implications

The application of P fertilizers to agricultural land, particularly in areas with P-deficient soils, could improve crop production and maintain soil fertility [1]. However, in case of low use efficiency, P will accumulate in soils that can be potentially harmful to the environment. In Loses Plateau, P use efficiency is lower in apple orchard (4–6%) than in farmland (10–15%), resulting in P accumulation in soils. Enhanced P losses through subsurface runoff on loam soils could be expected when Olsen-P is above 60 mg kg^−1^ in UK [15] and 22 mg kg^−1^ in Northern Ireland [16]. Soil would attain 25% soil saturation with P in 10–15 years, resulting in excessive runoff P concentrations in Netherlands [1]. Heckrath et al. [15] proposed a leaching change-point to predict the leaching potential of soil P, and found a clear change-point in the response curve of Olsen-P *vs*. CaCl_2_-P. This curve is very useful for estimating the amount of P needed to enhance Olsen-P to the critical level. In this study, a change-point of 46.1 mg kg^−1^ was observed in the in the response curve of Olsen-P *vs*. CaCl_2_-P (Fig 3), which was slightly higher than that of Yangling (39.9 mg kg^−1^), but far lower than that of Qiyang (90.2 mg kg^−1^) [17]. CaCl_2_-P above the change-point increased dramatically with increasing soil Olsen-P (slope = 0.0251), which was about 7 times that below the change-point (slope = 0.0036). This clearly indicates that the risk of P losses, especially via runoff, will increased sharply when Olsen-P content is above the change point. Therefore, it is imperative to optimize soil P management strategy to achieve optimal crop yield on one hand, and decrease soil P accumulation and losses on the other hand. The critical Olsen-P level for apple orchard in Loess Plateau is determined to be 15 mg kg^−1^, below which apple trees may suffer from poor growth. In this study, Olsen-P in apple orchards (19.6 mg kg^−1^ to 38.9 mg kg^−1^) was within the change points (15 mg kg^−1^ to 46.1 mg kg^−1^). If surplus P is applied, it will accelerate P accumulation in soil and environment pollution.

### Uncertainty of calculate in P balance

Phosphorus in root is important for the estimation of P uptake by apple trees. However, it is difficult to collect the total root biomass in the field conditions, and P in root ultimately returns to soil through root decomposition. The lack of P in root contributes to the uncertainty in the estimation of P balance. The sampling strategy, space substitute for time, was used in this study. Moreover, a large number of samples were collected to reduce the error in the estimation of P balance. The total P in organs was equal to the biomass multiplied by the P concentration, where the same P concentration was used in all cases because there was no significant different in the P concentration of organs among different apple orchards (Table 1).

Apple trees have different P uptake capacities at different ages, and younger apple trees generally have stronger uptake capacities than old apple trees [41, 42]. However, in this study, there was no significant difference in P concentration in organs in different apple orchards except the branches (Table 1). Another factor for the uncertainty in P balance was the annual variation of temperature, precipitation and other factors. For example, apple trees could uptake more P in wet years than in dry years due to more rain water supply.

## Conclusions

Long-term application of surplus P fertilizers resulted in significant P accumulation in soils of apple orchards in Loess Plateau, China. As compared with the grassland, TSP was increased by 60% and Olsen-P was increased by 12 times (38.9 mg kg^−1^) in 15-year-old apple orchard. *K* and *Q*_*m*_ values decreased dramatically; whereas DPS increased with increasing P concentration. CaCl_2_-P increased significantly with the increase of Olsen-P, especially above the change point of 46.1 mg kg^−1^. Thus, surplus P may pose a significant environmental risk.

## Supporting Information

S1 Table (Relationship between total soil P and Olsen-P in P-accumulated soils). (DOC)

S2 Table (Relationships between CaCl_2_-P and Olsen-P in P-accumulated soils). (DOC)

S3 Table (Relationships among K-value, Qm, DPS, and CaCl_2_-P under P-accumulated soils). (DOC)

S4 Table (Total P and Olsen-P distribution in the 0 − 200 cm profile under P-accumulated soil). (DOC)
