# Supplementary material for: Phosphorus Accumulation and Sorption in Calcareous Soil under Long-Term Fertilization
Source: PLoS One. 2015 Aug 19;10(8):e0135160. doi: 10.1371/journal.pone.0135160 (PMC4545939; doi:10.1371/journal.pone.0135160)
Supplement: S1 Table — (DOC) [file pone.0135160.s001.doc]

| TSP | Olsen-P | TSP | Olsen-P | TSP | Olsen-P |
| --- | --- | --- | --- | --- | --- |
| 710.00 | 25.35 | 870.00 | 35.87 | 617.17 | 6.06 |
| 750.00 | 22.38 | 880.00 | 70.39 | 630.00 | 3.08 |
| 740.00 | 17.96 | 932.13 | 78.67 | 660.00 | 8.97 |
| 814.76 | 42.13 | 940.00 | 51.66 | 680.00 | 12.30 |
| 720.00 | 21.14 | 970.00 | 63.72 | 710.00 | 13.77 |
| 704.59 | 6.90 | 1220.00 | 48.28 | 750.00 | 18.77 |
| 570.00 | 12.45 | 620.00 | 13.27 | 817.57 | 27.20 |
| 650.00 | 18.19 | 639.97 | 15.19 | 820.00 | 47.80 |
| 653.31 | 14.57 | 670.88 | 16.03 | 835.22 | 33.53 |
| 691.13 | 8.95 | 776.28 | 32.63 | 840.00 | 22.70 |
| 628.79 | 2.00 | 850.00 | 30.20 | 860.00 | 21.42 |
| 650.00 | 9.31 | 847.60 | 31.37 | 860.00 | 38.52 |
| 670.00 | 13.78 | 1223.22 | 34.15 | 880.00 | 30.86 |
| 698.61 | 9.50 | 780.00 | 32.25 | 930.00 | 36.66 |
| 790.00 | 28.45 | 920.65 | 43.50 | 950.00 | 34.83 |
| 660.00 | 12.46 | 847.90 | 53.35 | 610.00 | 2.17 |
| 694.73 | 14.85 | 988.01 | 43.29 | 1001.00 | 42.27 |
| 1011.39 | 85.40 | 620.00 | 10.48 | 1050.00 | 27.77 |
| 650.00 | 17.14 | 820.00 | 20.22 | 1460.00 | 53.08 |
| 680.90 | 18.42 | 1090.00 | 54.24 | 570.00 | 2.20 |
| 740.00 | 21.13 | 610.00 | 7.59 | 729.72 | 29.55 |
| 740.00 | 29.92 | 830.00 | 46.67 | 540.00 | 2.53 |
| 742.90 | 25.32 | 620.00 | 15.85 | 820.00 | 45.51 |
| 745.04 | 44.16 | 630.00 | 6.01 | 826.94 | 41.06 |
| 800.00 | 30.50 | 750.21 | 30.22 | 569.09 | 9.09 |
| 810.00 | 44.00 | 763.14 | 53.50 | 820.00 | 20.10 |
| 830.00 | 42.95 | 944.85 | 28.75 | 900.00 | 31.13 |
| 1010.00 | 64.20 | 660.00 | 13.88 | 691.72 | 11.48 |
| 633.73 | 12.08 | 837.19 | 43.14 | 760.00 | 47.72 |
| 650.00 | 10.15 | 656.78 | 7.76 | 760.00 | 20.36 |
| 720.00 | 30.66 | 760.00 | 22.68 | 800.00 | 67.09 |
| 730.00 | 26.24 | 1180.00 | 91.79 | 750.00 | 25.19 |
| 740.00 | 27.61 | 862.59 | 48.29 |  |  |
