# Supplementary material for: Phosphorus Accumulation and Sorption in Calcareous Soil under Long-Term Fertilization
Source: PLoS One. 2015 Aug 19;10(8):e0135160. doi: 10.1371/journal.pone.0135160 (PMC4545939; doi:10.1371/journal.pone.0135160)
Supplement: S2 Table — (DOC) [file pone.0135160.s002.doc]

| Olsen-P | CaCl2-P | Olsen-P | CaCl2-P |
| --- | --- | --- | --- |
| 2.17 | 0.14 | 29.551 | 0.087 |
| 2.2 | 0.118 | 32.634 | 0.168 |
| 3.08 | 0.118 | 33.528 | 0.04 |
| 6.06 | 0.042 | 41.058 | 0.358 |
| 8.97 | 0.197 | 42.267 | 0.118 |
| 9.501 | 0.283 | 43.136 | 0.586 |
| 11.48 | 0.103 | 43.287 | 0.115 |
| 12.076 | 0.165 | 43.497 | 0.413 |
| 12.46 | 0.127 | 47.8 | 0.289 |
| 13.17 | 0.14 | 48.29 | 0.649 |
| 13.77 | 0.165 | 53.08 | 0.124 |
| 14.57 | 0.121 | 53.504 | 0.443 |
| 14.846 | 0.258 | 78.666 | 1.019 |
| 16.03 | 0.118 | 85.396 | 1.352 |
| 18.77 | 0.246 | 28.45 | 0.137 |
| 27.197 | 0.146 | 28.747 | 0.184 |
