# Supplementary material for: Phosphorus Accumulation and Sorption in Calcareous Soil under Long-Term Fertilization
Source: PLoS One. 2015 Aug 19;10(8):e0135160. doi: 10.1371/journal.pone.0135160 (PMC4545939; doi:10.1371/journal.pone.0135160)
Supplement: S3 Table — (DOC) [file pone.0135160.s003.doc]

| CaCl_2_-P | K | Qm | DPS |
| --- | --- | --- | --- |
| 0.38 | 4.30E-03 | 909.1 | 1.6 |
| 0.52 | 6.50E-03 | 666.7 | 4.3 |
| 0.6 | 7.20E-03 | 434.8 | 7.9 |
| 0.89 | 4.20E-03 | 714.3 | 7.2 |
| 0.93 | 5.70E-03 | 400 | 13.4 |
| 1.29 | 2.80E-03 | 500 | 13.6 |
| 2.16 | 3.90E-03 | 434.8 | 20.8 |
