# Supplementary material for: Phosphorus Accumulation and Sorption in Calcareous Soil under Long-Term Fertilization
Source: PLoS One. 2015 Aug 19;10(8):e0135160. doi: 10.1371/journal.pone.0135160 (PMC4545939; doi:10.1371/journal.pone.0135160)
Supplement: S4 Table — (DOC) [file pone.0135160.s004.doc]

| depth | TSP(15-year) | TSP(10-year) | TSP(5-year) | TSP(Grass land) | Olsen-P(15-year) | Olsen-P(10-year) | Olsen-P(5-year) | Olsen-P(Grass land) |
| --- | --- | --- | --- | --- | --- | --- | --- | --- |
| 20 | 1340.00 | 860.00 | 760.00 | 538.78 | 86.38 | 20.92 | 13.92 | 1.73 |
| 40 | 980.00 | 770.00 | 700.00 | 515.29 | 42.68 | 11.66 | 6.26 | 0.56 |
| 60 | 700.00 | 680.00 | 610.00 | 544.24 | 9.05 | 3.49 | 2.34 | 0.44 |
| 80 | 640.00 | 630.00 | 610.00 | 546.85 | 4.44 | 1.45 | 1.25 | 1.79 |
| 100 | 640.00 | 580.00 | 640.00 | 563.21 | 6.37 | 1.14 | 1.38 | 0.55 |
| 120 | 640.00 | 580.00 | 640.00 | 553.31 | 8.57 | 1.25 | 1.48 | 0.47 |
| 140 | 630.00 | 600.00 | 620.00 | 555.09 | 7.70 | 1.55 | 1.22 | 0.80 |
| 160 | 660.00 | 590.00 | 600.00 | 576.42 | 7.72 | 1.07 | 1.26 | 0.56 |
| 180 | 650.00 | 620.00 | 590.00 | 611.28 | 9.19 | 2.62 | 1.26 | 0.55 |
| 200 | 660.00 | 620.00 | 580.00 | 621.44 | 9.50 | 4.81 | 1.36 | - |
